# Composition Profiles at the Metal Substrate–Deposit Interface Produced in Laser-Assisted Additive Manufacturing Processes

**DOI:** 10.3390/ma17133125

**Published:** 2024-06-26

**Authors:** László Péter, Szilvia Kugler, Tamás Kolonits, Attila Nagy

**Affiliations:** 1 HUN-REN Wigner Research Centre for Physics, P.O. Box 49, H-1525 Budapest, Hungary; nagy.attila@wigner.hun-ren.hu; 2 HUN-REN Centre for Energy Research, P.O. Box 49, H-1525 Budapest, Hungary; kugler.szilvia@ek.hun-ren.hu (S.K.); kolonits.tamas@ek.hun-ren.hu (T.K.)

**Keywords:** additive manufacturing, laser cladding, cross-sectional composition profile, asymmetric component distribution, remelting zone, intermixing layer

## Abstract

The cross-section of various substrate–deposit metal pairs obtained with a laser-assisted additive manufacturing process has been studied by observing the composition profile with energy-dispersive spectroscopy (EDS). The EDS composition profiles observed with a sufficiently high data acquisition time revealed that the composition profile is asymmetric. By scanning toward the growth direction, a sudden composition variation was observed, which was followed by a slow decay. The character of the composition profile was the same for a number of substrate–deposit pairs, and similar trends were found in various earlier publications as well. A mathematical model for the composition variation is suggested based on the assumption that a spontaneous homogenization process takes place in the intermixing (dilution) zone of the remelted top layer of the substrate. The equation obtained makes it possible to quantitatively describe the composition profile of each component that exhibits a concentration difference between the substrate and the deposit, provided that the mole fraction difference much exceeds the scattering of the data measured. The suggested model has also been applied successfully to composition profiles published in other works, hence exhibiting general relevance. Since the variation in some physical parameters (such as hardness) along the growth direction has been reported to follow the same pattern, it is assumed that the root cause in these cases may also be the composition variation.

## 1. Introduction

Laser cladding is an additive manufacturing (AM) process that can be used to add coatings onto a substrate to enhance its corrosion resistance, fatigue or wear properties, repair damaged surfaces or produce 3D parts layer by layer. The physical, mechanical and microstructural properties of 3D-printed metal parts have been extensively studied and reviewed [1,2,3,4,5,6,7,8].

An increasing interest is also raised in the characterization of the microphysical properties of the substrate–deposit interface. Different mechanical and bonding properties are desired at the interface between the substrate and the coating, depending on the goal of the AM process. A solid bonding is a must for hardness-enhancing or corrosion-inhibiting layers, as opposed to the case when a 3D object is created that later needs to be detached. For the laser cladding directed energy deposition (DED) additive manufacturing process, the hardness and the microstructure properties of the interface depend on the physical and chemical properties of both the applied alloy and the parameters of the cladding process, such as laser power, defocusing distance, scanning speed, powder feed rate and direction as well as shielding gas flow rate [9,10,11,12,13,14,15]. These parameters also play an important role in other techniques, like additive manufacturing of functionally graded structures [16] or large-scale additive manufacturing [17].

It is often desired that a gradient interface is formed between the substrate and the deposit [18,19,20,21], and the transition in such structures is intensively studied. Examples range from cases when a single-component interlayer is applied between dissimilar bulk materials [12,22,23] and samples with a gradual transition from one composition to another in minor steps [24,25,26,27,28]. Such intentional transients are usually applied with the purpose of establishing a solid bonding between metals having different thermo-physical properties. Although the bonding, the corrosion resistance properties, the hardness and the microstructure transition have already been investigated in several studies, these studies are not always connected to the investigation of the melt produced during the deposition. It is known from both experimental [29] and theoretical [30] works that the flow pattern produced in the molten pool during the laser cladding process has a symmetrical profile in the plane perpendicular to the motion direction of the laser. This melt flow is primarily responsible for the materials transport during the laser-assisted AM process. Both experimental and theoretical works concluded that the local flow velocity can reach the level of 1 m/s.

Various mathematical models are available for the formation of laser-cladded workpieces (e.g., [31,32,33]). The models handle lots of physical parameters such as the laser intensity distribution, the laser light–metal interaction and the thermodynamic and transport properties of the materials involved. However, these models are essentially physical in nature and do not deal with the elemental distribution at the substrate–deposit interface, even though other works deal with composition variation issues from both experimental and theoretical points of view [34,35].

In our work, the primary goal was the study of the solid deposit produced in the laser-assisted AM process, and as a part of this comprehensive study, we have characterized the composition transition at the substrate–deposit interface between four different metal alloys and stainless steel (as the substrate) by means of scanning electron microscopy (SEM) and energy-dispersive spectroscopy (EDS). This led to the observation of the asymmetric composition profile. The finding was occasionally checked with transmission electron microscopy (TEM). We have also introduced a mathematical model that describes the elemental concentration profile through the substrate–clad interface, which shows a very good agreement with the experimental results of the SEM-EDS line-scan measurements on the water-cut cross-section of our laser-cladded samples. For the sake of both the smooth understanding and the prompt comparison of the experimental result with the model predictions, the model is presented first, followed by the composition profiles measured. Based on our own findings, the scrutiny of the relevant literature revealed that similar asymmetric composition profiles have already been published without addressing the possible reasons for the formation of such interfaces. In addition, the strength and/or hardness profiles found in various earlier papers are qualitatively similar, giving a hint that the possible hidden reason for such a behavior might be sought in the composition evolution.

## 2. Materials and Methods

### 2.1. Materials

For the experiments, we used 304L (1.4307) stainless steel substrate in the form of 10 mm thick plates to deposit various coatings. Four different alloys were selected for the cladding in the form of powder (H13, 15-5PH, In625, In718). The compositions of the powders were in agreement with the relevant standards [36,37,38,39], as also confirmed by our own SEM-EDS analysis.

### 2.2. Additive Manufacturing Process

A 450 W fiber laser-based welding machine (LRS EVO-Diodeline 450) equipped with a coaxial powder nozzle (manufactured by the OR Laser GmbH, Dieburg, Germany, now part of Coherent, Saxonburg, PA, USA) was used for the cladding process to produce the samples. The single-mode diode-pumped ytterbium laser used has a wavelength of 1070 nm, and the beam reached the sample perpendicular to the base plate. The laser power per surface can be adjusted between 10% and full power, while the spot size may vary between 0.48 and 3 mm. The sample clads were 15 × 15 × 5 mm^3^ (width × length × height) rectangular cuboids on the above-mentioned substrate plates. The samples were built in an inert gas atmosphere (Argon 4.6 was the shielding and the carrier gas). The system was fed by a GTV PF2/1 LC powder feeder (GTV GmbH, Luckenbach, Germany) with the metal powders at a 5.3 g/min powder delivery rate and with a 3.8 L/min carrier gas flow rate. The nominal powder size was 45–90 µm for each starting material (which was also confirmed with SEM). While the scan speed and the laser spot size were fixed to 8 mm/s and 1.04 mm, respectively, the material-dependent optimal laser power and the resulting layer height achieved with a single scan were determined experimentally for the four alloys. The latter values are given in Table 1.

### 2.3. Sample Cutting and Polishing

In order to study the composition profile between the substrate and the cladding, the substrate plates were sliced into columns of 20 × 20 mm^2^ base surface area around the sample clads, and then each of them was cut into two parts perpendicular to the substrate–deposit interface with a water-jet cutting machine. When the transition zone between the substrate and the deposited material was in the range of 100 μm, the surface obtained after the water-jet cutting procedure was smooth enough for the observation of the composition profile.

Another procedure had to be followed when the transition zone exhibited a thickness of about 20 μm only. After cutting, the samples were also polished on a mechanical polishing bench with a Buehler diamond paste of gradually decreasing grain size until a mirror-like surface was obtained by visual observation. The polishing procedure was followed with an optical microscope. In most cases, the visually detectable interface between the substrate and the deposit disappeared by the end of the polishing procedure. The final polishing direction was parallel to the substrate–deposit interface in order to avoid the contamination of the sample surface from the polishing debris of either of the sample parts (substrate or laser-cladded deposit).

After cutting or polishing, the specimens were not exposed to any metallographic etching since the study of the phase structure and grain formation was out of the scope of the present study, but the original composition was intended to be conserved.

### 2.4. Scanning Electron Microscopy and Line Scan Measurements

A MIRA3 scanning electron microscope (TESCAN, Brno, Czech Republic) was used to image the cross-sectionally cut surfaces. Energy-dispersive X-ray analysis was performed with an EDAX Element analysis system (EDAX Inc., Mahwah, NJ, USA). During the analysis, the acceleration voltage was 20 kV. Line scans composed of 50 to 70 points were recorded for 10 to 35 min. The relatively long data acquisition time was particularly important in providing a sufficiently large signal-to-noise ratio, which is a prerequisite for quantitative analysis. Peculiar attention was paid so that the direction of the line scans was perpendicular to the substrate–deposit interface. It was also carefully checked that the spectra obtained did not vary with the sample position, especially its rotation, which excludes any artifact originating from the relative position of the interface and the detector axis. The spectra obtained for each point of the line scans were evaluated with the help of the instrument software (that automatically applies ZAF corrections; Element software version # 2.2.0001.0001).

### 2.5. TEM Sample Preparation and TEM Measurements

EDS line scan results for the 15-5PH sample were obtained by another SEM instrument. For these measurements, a Thermo Fisher™ Scios™2 DualBeam™ scanning electron microscope was used (manufacturer: Thermo Fisher Scientific, Waltham, MA, USA), operated at a beam voltage of 30 kV and a beam current of 3.2 nA. These measurements were made to confirm the earlier observation and select the area for the TEM sample preparation.

For TEM measurements, a lamella was cut from the sample, and it was thinned by a focused beam of Ga^+^ ions in a ±20 μm range of the interface. The measurements were taken with a Cs-corrected Themis-200 (S)TEM operated with 200 kV accelerating voltage. EDS mappings were acquired with Super-X EDX detectors in STEM mode (manufacturer of both the TEM and EDS instruments: Thermo Fisher Scientific, Waltham, MA, USA). With the selected high beam current, the point resolution that could be achieved was as small as 0.2 nm; however, the pixel size of the STEM images was ~3.2 nm. Therefore, this latter value restricted the lateral resolution of the EDS elemental mappings. In addition, to achieve good statistics in a reasonable time, STEM EDS signals were averaged for 32-pixel-height (approximately 102 nm) regions.

## 3. Results

### 3.1. Theory: Modeling the Composition Transition

**Figure 1:**
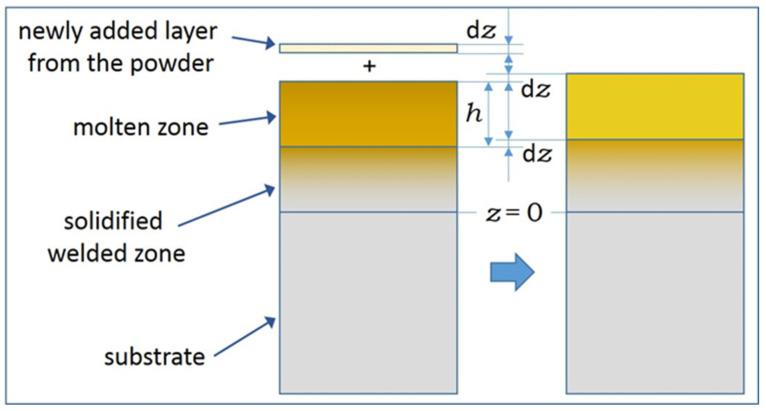
Two stages of the solidification near the interface of the substrate and the welded part. A partial remelting of the substrate and the concomitant change of the molten zone composition, as well as its displacement upon the newly added material, are considered.

A calculation mode is hereby suggested for the assessment of the concentration profile around the substrate–welded part interface if the partial remelting of the substrate is considered. Remelting is commonly considered in laser welding processes (see, e.g., Figure 5 in [40]; also [29,30,41]). Figure 1 shows two stages during the advancement of the welding process and indicates the notations as well.

It is assumed that the thickness of the molten zone (h) is constant, and the melt is compositionally homogeneous. The growth of the welded part takes place in a manner so that the newly formed solid layer is of the same composition as the melt itself. This means a non-equilibrium solidification since the equilibrium of solid and molten metals usually takes place at the dissimilar chemical composition of the phases. Other simplifying conditions applied for the calculation were as follows: (i) The molar volume of all components is identical; (ii) the remelted zone together with the newly packed deposit is fully homogenized and (iii) no significant solid-state diffusion takes place. The above approximation corresponds to a situation where the primary driving force of concentration changes is independent of the components. This can be convection, which is rationalized by the large flow velocity occurring in such a situation [29,30]. When the powder is fed constantly to the welding spot, the impact of the incoming particles can also contribute to the mechanical forces. The prerequisite for this situation is that the mechanical forces leading to convection are strong enough, but the solidification is also sufficiently fast so that the diffusive transport of the components is negligible. These conditions are customary for the moving speed of the laser (several cm/s or higher). The structural aspects of the solidification are not scrutinized here; the only aspect studied is the composition profile.

The conditions applied in the present model are partly identical to those used in previous approaches. For instance, in Scheil’s model for the solidification of binary alloys [42], the solidification is unidirectional, and the diffusion in the solid phase is also neglected. An important difference is that Scheil attributed the homogenization of the liquid to diffusion, not to mechanical forces. While in Schiel’s approach, a local equilibrium at the phase boundary is applied with a certain partition coefficient, our model does not assume an equilibrium solidification. In this respect, the model we propose is rather similar for melt quenching than equilibrium solidification.

Assume that the addition of the metal powder during the cladding process thickens the melt with dz at its open surface. In parallel, the solid part also grows with the same thickness by displacing the solidification front to the same extent, hence leading to a molten zone of constant thickness. Now write the mole fraction (x) of component i of the melt as the front moves with dz:

(1) $$x_{i}\left(z+\mathrm{dz}\right)=\frac{x_{i}\left(z\right)\left(h-\mathrm{dz}\right)+x_{i}^{\prime }\mathrm{dz}}{h},$$

where x′ refers to the mole fraction of component i in the powder used for cladding.

After a short rearrangement:

(2) $$\frac{dx_{i}}{\mathrm{dz}}=\frac{1}{h}\left[x_{i}^{\prime }-x_{i}\left(z\right)\right].$$

By using

(3) $$∆x_{i}=x_{i}\left(z\right)-x_{i}^{\prime }$$

one obtains that

(4) $$\frac{d{∆x}_{i}}{\mathrm{dz}}=-\frac{1}{h}{∆x}_{i}$$

which obviously leads to

(5) $$∆x_{i}=\mathrm{Kexp}(-z/h)$$

where K is an integration constant that is to be eliminated from initial and boundary conditions. If the boundary of the never-melted part of the substrate is at z = 0,

(6) $$∆x_{i}=x_{i}^{*}-x_{i}^{\prime }$$

where $x_{i}^{*}$ is the mole fraction of the same component in the substrate. Hence, the final solution is as follows:

(7) $$X=\frac{x\left(z\right)-x_{i}^{\prime }}{x_{i}^{*}-x_{i}^{\prime }}=\exp(-z/h)$$

which means that the decay rate of the concentration is expected to be solely a function of the thickness of the remelted zone. The relative mole fraction change X, as defined by Equation (7), must be identical for all components since we have not made any preliminary assumption about it (major component or trace element, increasing or decreasing concentration). Since the entire approximation is based on complete intermixing in the molten state during the transport (i.e., convection or any other non-specific process that does not discriminate components, unlike diffusion), this uniformity is well expected.

It is to be stressed that the approach described above also works in the case when the molten zone is significantly thicker than its near-substrate boundary in which the intermixing of the components can indeed take place (the latter is often called the dilution zone). If the intermixing zone is a thin layer only in the middle of the molten layer, but the homogenization condition within this zone holds, the equations describing the composition profile remain the same. Hence, it is expected that the same composition profile can be obtained for a wide range of deposits, and the h parameter in the equations above will mean a lower limit only for the thickness of the molten zone.

It is to be noted that an exponential decay may also be produced during the equilibrium solidification of alloys [43,44]. However, in the works cited, the transient is due to the partition of the components between the solid and the liquid, and the formation of the new solid layer does not take place on a substrate of dissimilar composition whose remelted part might affect the composition transient.

### 3.2. Experimental Composition Profiles

The boundary line between the substrate and the welded deposit could not be seen in the secondary electron images after either the water-jet cutting or the fine polishing (see Supplement, Figures S1 and S2). The transient zone could be established from the EDS line scan only. The same was found for the backscattered electron images when the change in the mean atomic number is small, and no segregation is produced during the welding process (H13, 15-5PH, In625).

**Figure 2:**
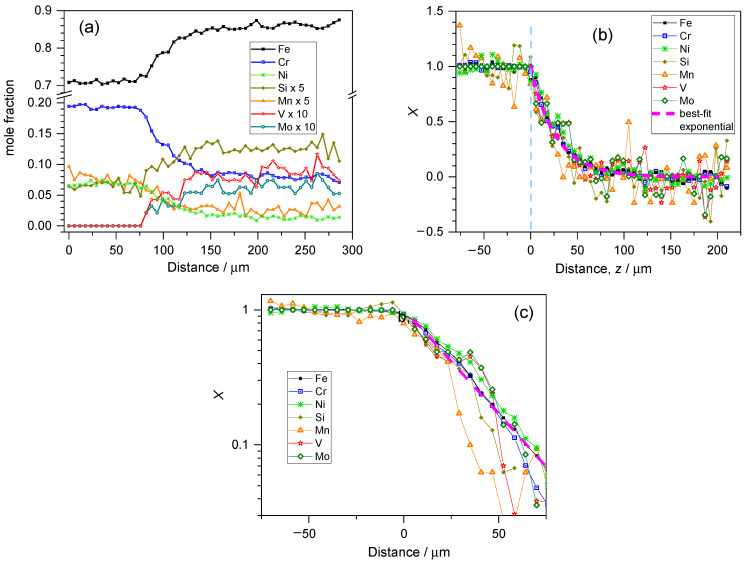
(**a**) Quantitative composition profile of the line scan shown in Figure S1 for a deposit produced with H13-type powder. The origin of this graph corresponds to the bottom end of the scan line (substrate side). (**b**) Evaluation of the composition profiles in accordance with Equation (7). The vertical segmented light blue line indicates the substrate–deposit boundary, and the abscissa is recalculated accordingly. (**c**) Semi-logarithmic plot of the 3-point moving average of the data shown in (**a**,**b**). The trend line is marked with a thick dashed line in (**b**,**c**) for the deposit side of the interface.

Evaluation of the EDS spectra of the H13 sample (as transformed to mole fractions) obtained at each point of the line scan shown in Figure S1 results in the composition profile presented in Figure 2a. In all subsequent plots, the spatial coordinate increases from the substrate toward the deposit side. When these spectra are transformed in agreement with Equation (7), the graph presented in Figure 2b can be obtained. It is obvious that the decay of the concentration of all components follows the same trend, which can be estimated with the exponential function plotted on the same graph. Figure 2c shows the same data in a semi-logarithmic representation after smoothing.

Note here that the data can be displayed on the semi-logarithmic plot until the distance when the scattered deviation from the average exceeds the X value itself since negative X values also start to appear, which cannot be displayed in a logarithmic plot. Relative concentrations with X < 0 or X > 1 values obviously cannot have any physical meaning, but such data always appear due to the derivation of X in agreement with Equation (7). This is merely the consequence of the error of the measurement and the fluctuation of the concentrations measured around their mean value.

**Figure 3:**
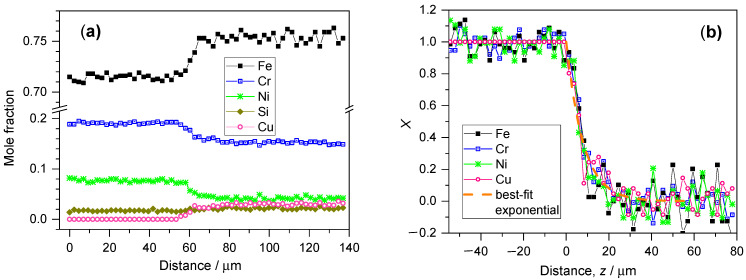
(**a**) Quantitative composition profile of the line scan for a deposit produced with 15-5PH-type powder. The origin of this graph corresponds to the bottom end of the scan line (substrate side). (**b**) Evaluation of the composition profile presented in graph (**a**), in accordance with Equation (7). The abscissa is recalculated so that the zero value falls to the interface between the substrate and the deposit.

The primary composition profile data obtained for a deposit made of 15-5PH-type metal powder is shown in Figure 3a, while the transformation of this dataset in accordance with Equation (7) is presented in Figure 3b. The primary composition profile dataset shows all elements whose concentration was above the detection limit of the EDS line scan analysis, but Si was omitted from the graph of the transformed data because the difference between the bulk substrate and deposit values in the Si mole fraction was negligible. The lack of data scattering for X_Cu_ in Figure 3b is because the Cu concentration was below the detection limit in the substrate.

A TEM sample was also prepared from the 15-5PH specimen to control the composition variation. Although the composition step could be established in the TEM EDS profile, the error of the composition determination was rather high. This was due to the undulation of the sample thickness and the smaller excited volume. The SEM and TEM composition profiles obtained on approximately the same area are presented in the Supplement (Figures S3 and S4). Since the TEM sample preparation means a further destructive sample preparation step, the SEM-EDS method proved to be superior for the observation of the composition profile, especially because repolishing the sample easily renews the surface and makes it possible to perform further observations.

**Figure 4:**
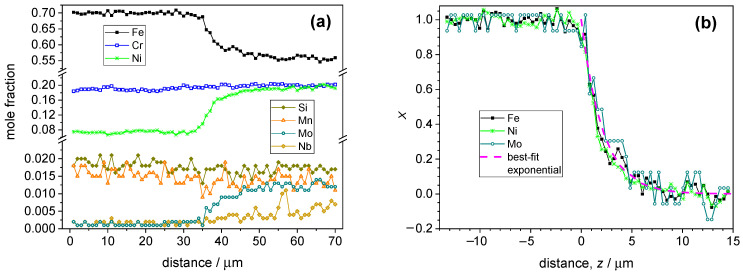
(**a**) Quantitative composition profile of the line scan for a deposit produced with the In625-type powder. (**b**) Evaluation of the composition profile presented in graph (**a**), in accordance with Equation (7). The abscissa is recalculated so that the zero value falls to the interface between the substrate and the deposit.

The quantitative depth profile obtained for the laser-cladded deposit with the In625 powder is shown in Figure 4. Figure 4a shows that the concentration of a few components (Cr, Si, Mn) is so close in the substrate and in the deposit that the zone boundary does not manifest itself in the distance dependence of their composition. Therefore, they are omitted from the subsequent analysis, and only the components with an unambiguous change in concentration are displayed in the corresponding X(z) graph (Figure 4b). The latter diagram shows that the concentration variation for Fe, Ni and Mo can be well described with the exponential function (Equation (7)). Niobium, while it was practically absent in the substrate, could be identified as a component of the deposit, but its concentration was not sufficiently large for a reliable analysis.

**Figure 5:**
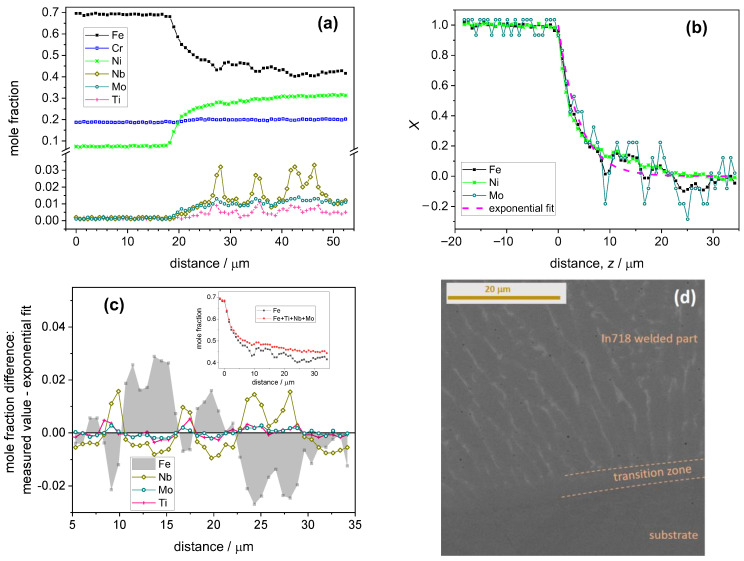
(**a**) Quantitative composition profile of the line scan for a deposit produced with In718-type powder. The origin of this graph corresponds to the bottom end of the scan line (substrate side). (**b**) Evaluation of the composition profile presented in graph a, in accordance with Equation (7). The abscissa is recalculated so that the zero value falls to the interface between the substrate and the deposit. (**c**) Difference between the measured mole fractions and those calculated with the help of the exponential fit as a function of the distance from the interface. The inset shows the comparison of the iron mole fraction and the sum of the mole fractions of the components that exhibit a correlated composition fluctuation. (**d**) Backscattered electron image of the cross-sectionally polished In718 sample with the identification of the main parts. The light strips in the welded part correspond to Nb segregations. For further data, see Supplement, Section S3.

Composition profiles for a deposit obtained with the In718 powder are shown in Figure 5a. Among the major components, the concentration change of Fe and Ni is large enough for the assessment of the decay rate parameter. However, the concentration of Cr was essentially identical in the substrate and in the deposit; hence, its concentration was omitted from the subsequent analysis. It is to be noted that the fluctuation of the Cr concentration was not correlated with any other parameter to be discussed below. Besides Fe and Ni, Mo was present in the deposit in a large enough concentration so that Equation (7) could be applied for its concentration decay. As it is shown in Figure 5b, one can obtain a uniform concentration decay pattern similar to the earlier samples.

In contrast to the earlier samples, the In718 coating shows new features not seen before. Namely, the concentration decay is not smooth for several alloy components, and the concentration fluctuations are highly correlated. The deviation of the local concentrations from those estimated by using Equation (7) is depicted in Figure 5c. This clearly shows that Mo, Ni and Ti are locally accumulated in a correlated manner, and their local accumulation is accompanied by a drop in the Fe mole fraction (and vice versa). As shown in the inset in Figure 5c, the sum of the concentration of these elements exhibits a much smoother decay than either of the concentration profile functions. The observed correlations in the local fluctuations of the concentrations of specific minority alloy components are attributed to component segregation, which is shown in Figure 5d. Line strips in the backscattered electron image indicate niobium-rich zones. The segregation-rich part is just beyond the transition zone, in which about 4/5 part of the composition decay takes place. Further data on the analysis of the segregation can be seen in the Supplement (Section S3, Figures S5 and S6).

The visual impression of the composition profiles obtained for the various deposit materials suggests that the thickness of the transition zone varies significantly from one sample to another. The fitted decay parameters obtained together with their estimated scattering are summarized in Table 2. The dissimilarity of the h parameter can be explained, besides the difference in the composition, with the fact that the laser operation parameters varied in a wide range for ideal deposit formation, as shown in Table 1. Nevertheless, each h value is smaller than the typical particle size of the feed powders. This is easily understood if one assumes that all particles arriving at the surface melt instantaneously and spread out on the surface at a much larger area than their original diameter. The latter provides that the build-up of the layer follows the scheme that was assumed for the quantitative evaluation of the composition profile. It is also likely that the parameter h is the effective thickness of the intermixing zone that only gives a hint of the lower limit of the molten layer thickness.

## 4. Discussion of the Literature Data on Composition Profiles and Other Properties

The shape of the composition profile functions presented in Section 3.2 is not unique. Similar line scans have been published elsewhere, although the possibility of the physical description has not been recognized. Since the goal of the composition profiling measurement ranged, in many cases, at most to the semi-quantitative check of the result of the deposition process only, little or no attention was paid to the reduction in the signal-to-noise ratio. Nevertheless, the shape of the composition profile can be well identified in a number of studies.

**Figure 6:**
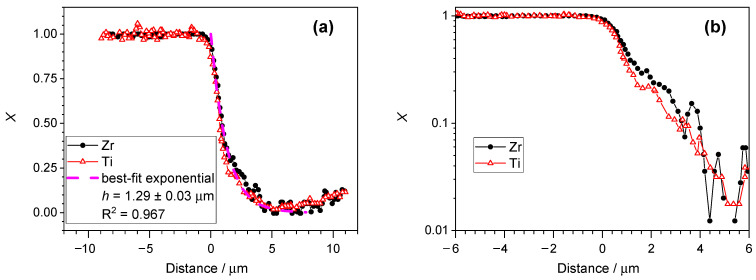
(**a**) Transform of the composition profile published by Li et al. for a Zr-rich deposit welded onto a Ti-6Al-4V substrate ([45], Figure 7b in the cited work). The origin was set to the interface between the substrate and the deposit. (**b**) Semi-logarithmic plot of the same data.

Figure 6 shows the transformation of the composition profile published by Li et al. [45] for laser welding of Zr_52.5_Ti_5_Al_10_Ni_14.6_Cu_17.9_ amorphous foils onto a Ti-6Al-4V substrate. The published composition profile only showed the major elements, Zr and Ti. The agreement with the prediction of Equation (7) is striking. The interface between the substrate and the deposit does not seem to be sharp for the data set adopted from [45]. The reason for the gradual composition change near the interface is probably due to the fact that the spatial frequency of the data acquisition (about 0.3 μm along the interface normal) was rather large, which means that the excitation volumes sampled with the EDS analysis in the neighboring data points overlapped significantly. The information volume of the EDS measurement is usually about 1 μm^3^ at 20 kV excitation. Therefore, the result is a convolution of the true composition profile with a relatively wide sampling width (or, in other words, with a sampling probability function of at least 1 μm width).

The analysis of the data shown above is particularly important since Li et al. [45] established both the thickness of the molten zone and the depth of penetration of the components of the same welded deposit. The distance between the unmodified deposit and the substrate was in the range of 100 μm, while the h parameter of the exponential fitting (Equation (7)) was only about 1.3 µm. This comparison clearly indicates that the composition decay can take place over a much shorter distance than the thickness of the molten zone, and the intermixing may be restricted to a relatively narrow molten layer (dilution zone) only.

Liu et al. study the transition between Ti6Al4V and Ti48Al2Cr2Nb alloys [46]. The peculiarity of this work is that the interface was formed between two laser-welded layers without the interruption of the AM process but changing the power feed line only. Although the scattering of the EDS data obtained in the cross-sectionally polished surface is quite scattered (see Figure 2 of the above-mentioned paper [46]), it can be well recognized that the concentration change rate decreases monotonously from the substrate side of the interface toward the bulk deposit. The interfacial concentration profile for the major components (Al and Ti) complies with the model proposed in the present work.

In the study of Li et al. [23], a Ti6Al4V → V → Cr → Fe → SS316 layer sequence was produced with a laser-based AM process (arrows indicate the growth direction). The layer boundaries can be well recognized in their EDS composition profile. Although not all transients obey a full regularity, several mole fraction transients appear to be in line with the exponential decay predicted in the present work (V and Cr at the V → Cr transition zone; Cr, Fe and V at the Cr → Fe transition zone; see Figure 5 in the above-mentioned work [23]). The decay of the mole fractions extends nearly to the millimeter range at each transient zone, as opposed to several studies cited above.

It is to be observed that the decay rate varies widely in various studies, even if the character of the composition transients is alike in the sense of asymmetry. The reason for such a variety in the composition decay rate may be a difference in the power density of the lasers used, the difference in the melt pool temperature as well as the incomparable viscosity and transport properties of the alloying elements. While the transient zone for each boundary can be assessed to be 150–200 μm wide in a Ti6Al4V → V → Cr → In718 graded sample [13], some 70–100 μm wide transition zone can be identified when a Ti6Al4V → Mo transition was produced in a step-wise manner [25]. The latter study is particularly interesting because, at the 0% → 25% and 25% → 50% Mo steps, the decay starts at the surface of the previously laid deposit, but the gradient looks to be inverted at the 75% → 100% Mo step. A similar inversion in the asymmetry of the composition decay can be seen in various alloy pairs in which the deposit is of significantly higher melting temperature than the substrate (Ti2AlNb → Ti6Al4V [47], Ti6Al4V → Ta [48]). Hence, an inversion in the order of solidification of the layers may deviate from the model proposed in the present work.

An exponential-like composition decay was also found for laser-based AM deposits intentionally graded at the millimeter scale with 316L substrate and In718 deposit [26]. In the latter study, when the composition transient was achieved gradually (i.e., in several fine grading steps with small variations in the powder composition), the composition gradient could be well fitted with an exponential function (Figure 7d of [26]). However, it must be stressed that this situation can occur only when the thickness of the intermixing zone is comparable to the deposit thickness layered in each step. On the contrary, when the dilution zone is much smaller than the thickness of the layers of even composition, an asymmetric profile ought to be obtained in each layer, as will be seen in the forthcoming example.

The asymmetry of the composition profiles was well identified in some cases when several layers of the deposits were laid onto each other on a substrate of dissimilar composition. In the study of Qian et al. [24], a successively varying composition was obtained in the subsequent layers, but the asymmetric profile can be identified in each layer in which the composition change is sufficiently large as compared to the previous layer (see the top part of Figure 5 of the above-mentioned study [24]).

Besides the composition depth profile, some literature data on the microhardness profile of laser-cladded metals show similar trends as the composition depth profiles discussed above. In the works of Alam et al. [49,50], a sudden drop of microhardness can be seen at the boundary of the heat-affected zone and the dilution zone, followed by an asymptotic decay toward the bead zone. The microhardness decay, while the structural properties of the metal are also of importance, may be affected by the composition variation. Interestingly, a similar microhardness variation can be seen at the heat-affected zone (which presumably does not melt during the laser cladding process); however, a solid-state diffusion may also occur, leading to similar composition profiles. Although a bit less clear, a sudden increase in the microhardness can be seen at the cladding side of the substrate–deposit interface in the work of Qian et al. (see the mean value of the microhardness profile in Figure 11a of [51]). Unfortunately, the authors are not familiar with any work where the composition and the microhardness profiles were measured simultaneously on identical samples. The direct comparison of these parameters would be essential to clarify to what extent the composition decay is responsible for the mechanical behavior of the laser-cladded samples.

## 5. Conclusions

The data of the composition profile obtained for a number of substrate–laser-cladded deposit systems show that the composition decay is asymmetric. By using the proper transformation method, the composition decay of all components (elements) follows the same trend, which can be described with an exponential function. The composition decay stems from the intermixing of the newly added layer and the remelted zone of the substrate. The solution of the corresponding differential equation revealed that the composition decay could be related to the thickness of the intermixing zone, which is a part of the molten metal layer. The simple yet effective model takes into account an essentially convective transport process that disregards the diffusive motion of the individual components, hence leading to a general expression that is obeyed by all components of a sample. The validity of the expressions obtained proved to be independent of the material qualities of the substrate–deposit pairs studied in the present work; moreover, it was found applicable to composition profile data published in other articles. Hence, the model proposed may have general validity and open a way for the quantitative analysis of the composition depth profiles. It is yet to be elaborated how the thickness of the dilution zone is affected by the elementary physicochemical parameters of the system (such as the deposit growth rate, molten pool temperature, heat conductivity of the substrate, viscosity of the molten metal and the solidification rate). A similar spatial variation in strength and/or hardness published in the AM literature without a simultaneous measurement of the corresponding composition profile has also been discussed in light of our results, emphasizing that the primary parameter behind this behavior can be a concomitant composition decay.

## Figures and Tables

**Table 1:** Laser cladding parameters of the samples.

Alloy	H13	15-5PH	In625	In718
Power [W]	382	360	382	382
Layer height [mm]	0.5	0.45	0.3	0.2

**Table 2:** Decay parameter h of composition variation at the interfaces as defined in Equation (7) and the statistical parameters of the exponential fitting.

Alloy	H13	15-5PH	In625	In718
h [μm]	27.1 ± 1.0	7.88 ± 0.41	2.10 ± 0.07	3.97 ± 0.17
R^2^	0.783	0.820	0.926	0.853
χ^2^	0.0019	0.0095	0.0044	0.0076

## Data Availability

The raw data supporting the conclusions of this article will be made available by the authors on request.

## Supplementary Materials

The following supporting information can be downloaded at https://www.mdpi.com/article/10.3390/ma17133125/s1, Figure S1: Experimental composition profile of the H13 sample as shown during the EDS line scan on the cross-section of the welded object. The sample was water-cut but not polished. The line scan was performed along the vertical dark green line in the centre of the image. The right axis shows the distance in arbitrary units (number of the measurement point), while the top axis corresponds to the integrated line intensity of the specific elements in the X-ray energy range of the major fluorescent lines of the components. The horizontal dashed line represents the substrate–deposit interface, while the arrow at the left indicates the distance parameter used in Equations 1 through 7. Figure S2: Experimental composition profile as shown in the EDS line scan on the cross-section of the welded In625 deposit. Since the composition transition zone was restricted to a much narrower band than in the H13 sample, the polishing step was indispensable. The sample was water-cut and then polished to a mirror finish. Other image features are the same as for Figure S1. Figure S3: (a) Quantitative composition profile of the welded 15-5HP deposit. (b) Transformed data of the graph in (a) with the exponential fit (h = 6.9 μm). Figure S4: Quantitative TEM EDS composition profile of the welded 15-5HP deposit. Figure S5: Experimental composition profile as shown in the EDS line scan on the cross-section of the welded In718 deposit across the substrate–deposit boundary where no segregation zone falls into the analysed region. Line scan was carried out along the tilted dark green line. Signal intensities correspond to the energy range of the major lines of each element. Figure S6: Experimental composition profile as shown in the EDS line scan on the cross-section of the welded In718 deposit within the welded zone approximately at a right angle to the axis of the segregated Nb-rich dendrites. Line scan was carried out along the tilted dark green line. Signal intensities correspond to the energy range of the major lines of each element.
