# Supplementary material for: Composition Profiles at the Metal Substrate–Deposit Interface Produced in Laser-Assisted Additive Manufacturing Processes
Source: Materials (Basel). 2024 Jun 26;17(13):3125. doi: 10.3390/ma17133125 (PMC11242667; doi:10.3390/ma17133125)
Supplement: Supplementary file 1 [file materials-17-03125-s001.zip › materials-2991508-supplementary.pdf]

*Supplementary material for:*

# Composition Profiles at the Metal Substrate–Deposit Interface Produced in Laser-Assisted Additive Manufacturing Processes

**László Péter, Szilvia Kugler, Tamás Kolonits and Attila Nagy**

### *S1. Direct experimental observation of the asymmetric depth profile on samples with various surface finishing*

The boundary between the substrate metal and the deposit was not visible either by naked eye, with an optical microscope or in the SEM secondary electron images. The figures in this section demonstrate that the line scans help position the boundary only either in the sample as obtained after the water-jet cutting (H13 sample, Figure S1) and after fine polishing (In625 sample, Figure S2).

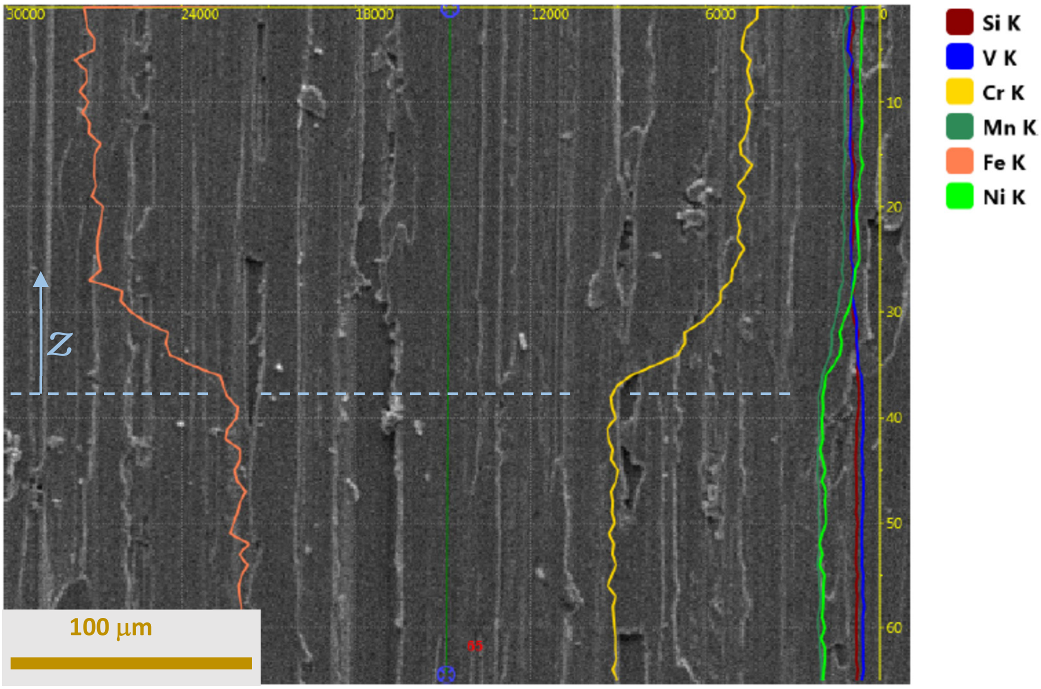


**Figure S1**. Experimental composition profile of the H13 sample as shown during the EDS line scan on the cross-section of the welded object. The sample was water-cut but not polished. The line scan was performed along the vertical dark green line in the centre of the image. The right axis shows the distance in arbitrary units (number of the measurement point), while the top axis corresponds to the integrated line intensity of the specific elements in the X-ray energy range of the major fluorescent lines of the components. The horizontal dashed line represents the substrate–deposit interface, while the arrow at the left indicates the distance parameter used in Equations 1 through 7.

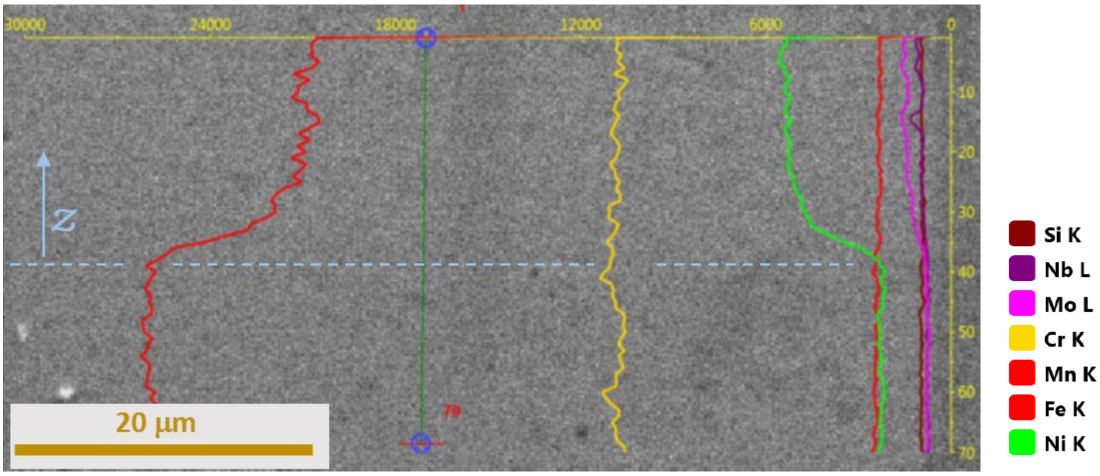


**Figure S2**. Experimental composition profile as shown in the EDS line scan on the cross-section of the welded In625 deposit. Since the composition transition zone was restricted to a much narrower band than in the H13 sample, the polishing step was indispensable. The sample was water-cut and then polished to a mirror finish. Other image features are the same as for Figure S1.

### *S2. Comparison of the SEM EDS and TEM EDS data for the 15-5HP sample*

Figure S3 show the SEM EDX line scan of a selected area of the 15-5HP samples. The two graphs in the latter figure display both the mole fraction vs. distance function and the transformed data with exponential fitting.

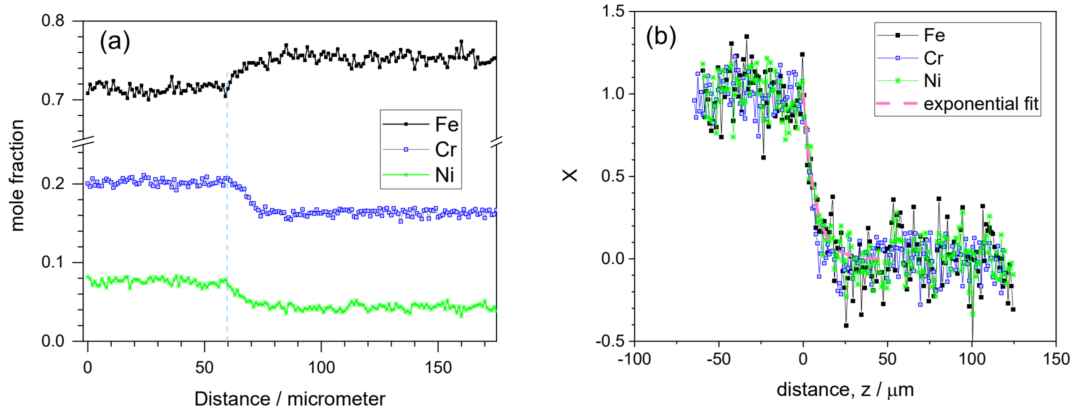


**Figure S3**. **(a)** Quantitative composition profile of the welded 15-5HP deposit. **(b)** Transformed data of the graph in (a) with the exponential fit (h = 6.9 µm).

TEM EDS line scan of the same region of the 15-5HP sample as scanned with the SEM EDS is shown in Figure S4. Due to the fact that relatively large sections had to be thinned, the sample thickness exhibited fluctuations (the sample produced appeared to be "wavy"). This had a large impact on the analysis result, leading to fluctuations that were not seen the SEM EDS data. Error of the results is typically ±4.5 at% for Fe (5 % of the mean value), 2.9 at% for Cr (14 % of the mean value) and 1.2 % for Ni (17 % of the mean value).

Since the TEM sample preparation is a time-consuming and very device-intensive technology, but the results may be influenced by artefacts, the SEM EDS analysis proved to be simple and repeatable at various areas with no limitation.

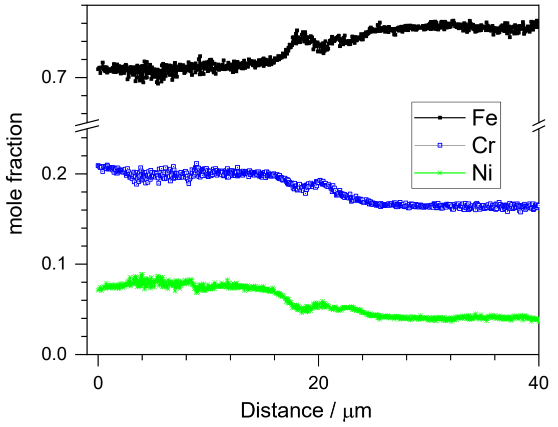


**Figure S4**. Quantitative TEM EDS composition profile of the welded 15-5HP deposit.

### *S3. Observation of the niobium segregation in the In718 sample*

For explaining the Nb fluctuation in the In718 sample, backscattered electron images were recorded for the cross-sectionally polished sample. Niobium segregations appeared in the welded zone, starting at a distance from the substrate where the composition transition essentially decays. Figure S5 shows the EDS line scan at a part where the scanning line does not cross the segregated Nb-rich zones.

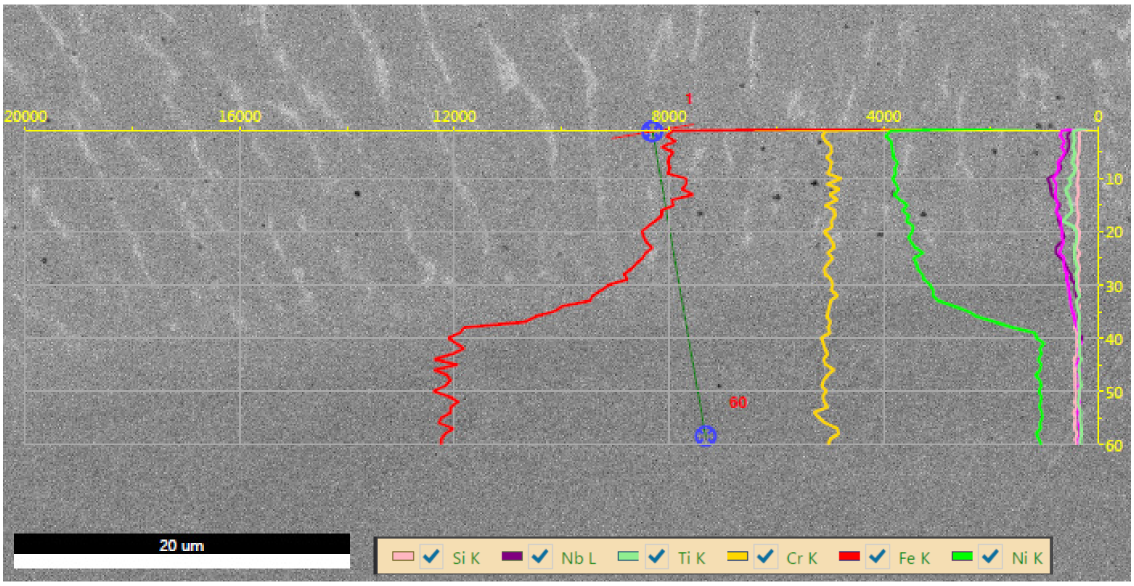


**Figure S5**. Experimental composition profile as shown in the EDS line scan on the cross-section of the welded In718 deposit across the substrate–deposit boundary where no segregation zone falls into the analysed region. Line scan was carried out along the tilted dark green line. Signal intensities correspond to the energy range of the major lines of each element.

Figure S6 shows a line scan performed within the welded zone. The direction of the line scan was chosen so that the elongated Nb-rich zones were crossed perpendicularly to their longitudinal axis. The strong intensity fluctuation of the Nb L line clearly indicated the Nb-rich segregation, and the concomitant counteroscillation of the Fe signal is also obvious.

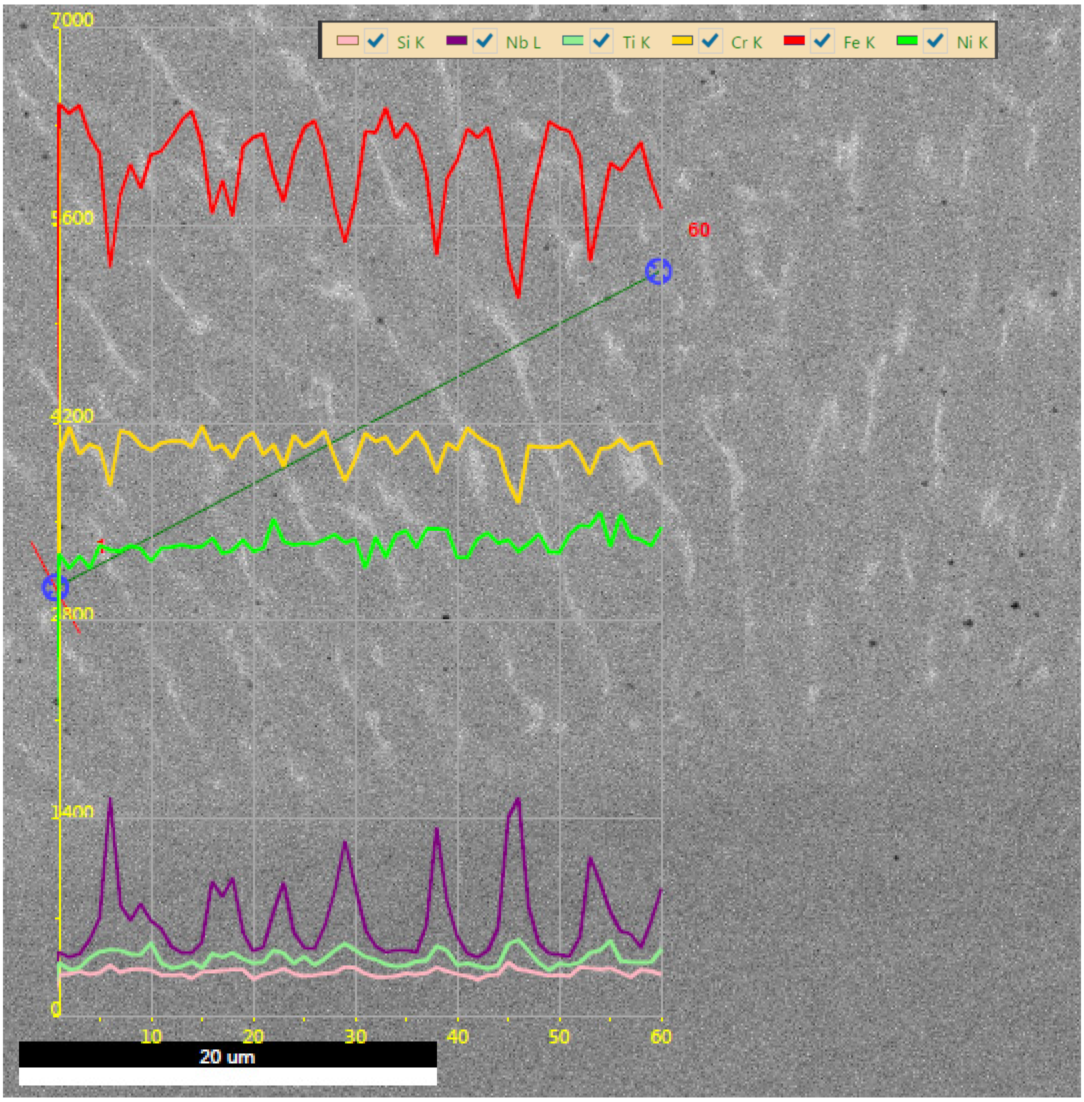


**Figure S6**. Experimental composition profile as shown in the EDS line scan on the cross-section of the welded In718 deposit within the welded zone approximately at a right angle to the axis of the segregated Nb-rich dendrites. Line scan was carried out along the tilted dark green line. Signal intensities correspond to the energy range of the major lines of each element.
